# Information for decision making from imperfect national data: tracking major changes in health care use in Kenya using geostatistics

**DOI:** 10.1186/1741-7015-5-37

**Published:** 2007-12-11

**Authors:** Peter W Gething, Abdisalan M Noor, Catherine A Goodman, Priscilla W Gikandi, Simon I Hay, Shahnaaz K Sharif, Peter M Atkinson, Robert W Snow

**Affiliations:** 1 School of Geography, University of Southampton, Highfield, Southampton, SO17 1BJ, UK; 2 Malaria Public Health & Epidemiology Group, Centre for Geographic Medicine, Kenya Medical Research Institute/Wellcome Trust Collaborative Programme, PO Box 43640, 00100 GPO, Nairobi, Kenya; 3 Consortium for Research on Equitable Health Systems (CREHS), Kenya Medical Research Institute/Wellcome Trust Collaborative Programme and London School of Hygiene and Tropical Medicine, PO Box 43640, 00100 GPO, Nairobi, Kenya; 4 Spatial Ecology and Epidemiology Group, Tinbergen Building, Department of Zoology, University of Oxford, South Parks Road, Oxford, OX1 3PS, UK; 5 Ministry of Health, P.O. Box 30016, Afya House, Cathedral Road, Nairobi, Kenya; 6 Centre for Tropical Medicine, University of Oxford, John Radcliffe Hospital, Headington, Oxford, OX3 9DU, UK

## Abstract

**Background:**

Most Ministries of Health across Africa invest substantial resources in some form of health management information system (HMIS) to coordinate the routine acquisition and compilation of monthly treatment and attendance records from health facilities nationwide. Despite the expense of these systems, poor data coverage means they are rarely, if ever, used to generate reliable evidence for decision makers. One critical weakness across Africa is the current lack of capacity to effectively monitor patterns of service use through time so that the impacts of changes in policy or service delivery can be evaluated. Here, we present a new approach that, for the first time, allows national changes in health service use during a time of major health policy change to be tracked reliably using imperfect data from a national HMIS.

**Methods:**

Monthly attendance records were obtained from the Kenyan HMIS for 1 271 government-run and 402 faith-based outpatient facilities nationwide between 1996 and 2004. A space-time geostatistical model was used to compensate for the large proportion of missing records caused by non-reporting health facilities, allowing robust estimation of monthly and annual use of services by outpatients during this period.

**Results:**

We were able to reconstruct robust time series of mean levels of outpatient utilisation of health facilities at the national level and for all six major provinces in Kenya. These plots revealed reliably for the first time a period of steady nationwide decline in the use of health facilities in Kenya between 1996 and 2002, followed by a dramatic increase from 2003. This pattern was consistent across different causes of attendance and was observed independently in each province.

**Conclusion:**

The methodological approach presented can compensate for missing records in health information systems to provide robust estimates of national patterns of outpatient service use. This represents the first such use of HMIS data and contributes to the resurrection of these hugely expensive but underused systems as national monitoring tools. Applying this approach to Kenya has yielded output with immediate potential to enhance the capacity of decision makers in monitoring nationwide patterns of service use and assessing the impact of changes in health policy and service delivery.

## Background

Driven by the international health agenda that supports the United Nations Millennium Development Goals, efforts are underway across Africa to improve access to health care and reduce barriers to service uptake [1,2]. Critical to the success of these efforts is the capacity of national governments to monitor effectively patterns of service use through time so that the impacts of changes in health policy or improvements in service delivery can be evaluated. Such capacity, however, is rare in Africa and other resource-constrained regions [3-5]. Existing efforts to monitor service use are often driven by international actors rather than national governments and are generally focused on specific interventions and age groups [6-8], limited to small geographical regions [9-11], or based on infrequently repeated national studies such as demographic and health surveys [12] that are insensitive to changes occurring over shorter timescales [13-15].

The importance of generating reliable statistics on variables such as service use is recognized by African governments, as demonstrated by the widespread uptake of assistance in statistical capacity-building offered by initiatives such as the Health Metrics Network [16]. Most Ministries of Health across Africa operate some form of health management information system (HMIS) as their primary instrument for generating health system statistics, often representing the majority of national expenditure on health data. Amongst other functions, HMISs generally coordinate the routine acquisition of monthly treatment and attendance records from health facilities nationwide and their compilation into a single national database. In principle, then, HMISs are the most appropriate and widely available tool for monitoring levels of service use within countries [17].

Despite the apparent suitability of an HMIS for monitoring a wide range of important health system metrics, and the substantial resources invested in their development and operation, the extent to which data from HMISs are used to generate statistics of use to decision makers is extremely limited [18]. The endemic under-use of hugely expensive HMIS data represents an unacceptable inefficiency in already resource-constrained health systems and can be attributed largely to the perceived unreliability of these data, due primarily to poor data coverage [19-21]. Typically, many health facilities never report monthly records to the HMIS, or do so only intermittently, leading to substantial gaps in national data [22,23]. Whilst international initiatives [16,24] to improve health information infrastructures in resource-constrained nations are to be welcomed, several decades of previous efforts by international donors have rarely yielded substantial improvements in HMIS data coverage [17,25].

In contrast to the widespread perception that existing HMIS data are inadequate for quantifying important health system metrics, recent studies have illustrated that such data can be used to answer certain questions reliably by employing appropriate statistical approaches [26-28]. In this study, we illustrate how levels of service use can be tracked reliably from incomplete HMIS data. We use a space-time geostatistical framework to account for the potential biases introduced by missing data and focus on the example of temporal changes in the use of clinics across Kenya between 1996 and 2004, a time of major changes in national health policy. In doing so, we aim to present a tool that can enhance capacity for evidence-based decision making using existing data and, more broadly, strengthen the case for renewed focus on the use of HMISs for health system planning and monitoring in resource constrained countries.

## Methods

### The Kenyan HMIS dataset

Data were obtained from the Department of HMIS of the Kenyan Ministry of Health. These routine HMIS data consisted of monthly records from outpatient departments of government and faith-based health facilities across Kenya. Data were available for a 108-month period from January 1996 to December 2004. As an indicator of service use, we extracted from each record the total number of new all-cause outpatient diagnoses made at a given facility during a given month. These values were not stratified by age or sex but excluded referrals and follow-up visits. Monthly outpatient data for each facility were matched to an independent database containing the spatial coordinates of government health facilities nationwide [29]. Ethical approval for this study was provided by the Kenya Medical Research Institute (KEMRI SSC 659).

### Estimating changes in service use through time

Several issues must be addressed if incomplete HMIS data are to be used to develop reliable time series showing patterns of service use through time. Given incomplete data, simply tallying the number of reported outpatient cases each month gives non-comparable national monthly totals since the number of facilities that report changes each month. As a straightforward way of standardising for this effect, we divided each monthly outpatient total by the number of facilities that reported in that month to create crudely standardised monthly and annual time series showing mean all-cause outpatient cases per facility per month.

Whilst the above approach accounts for the changing number of facilities reporting each month, the unknown influence of missing data from non-reporting facilities is ignored. Because the set of available data for a given month was likely to be from a non-random subset of facilities due to uneven reporting rates across the country, ignoring the large proportion of missing data could have led to biased estimates of mean outpatient cases per facility. A range of techniques have been developed across social, economic, and medical statistics to address problems of missing data, most notably the multiple imputation approach [30]. However, these techniques have been designed primarily to deal with multivariate datasets, with missing data on one variable for a given case or subject being predicted using information from other variables recorded for that case or subject [31]. In contrast, the present setting represents a univariate prediction problem: where a monthly outpatient record was missing from a given facility, no covariate information was available in our HMIS dataset to assist in predicting the unknown value. Although the class of each facility was known (e.g. hospital, health centre, dispensary), reliable and consistent nationwide data on service provision levels at each facility (such as number of beds and practitioners, services provided, financial data) were not available. However, the location in space (georeferenced coordinates of each facility) and time (indexed by month) of both data and missing data was known and this allowed the prediction problem to be set in a space-time modelling context, with separate models developed for each of the three facility classes.

Whilst multiple imputation approaches have been extended to address datasets constructed over time and/or across space [32-35], they remain reliant on the availability of covariates to predict missing values. The alternative paradigm of geostatistics is widely used in atmospheric [36,37] and Earth sciences [38,39], and more recently in public health and epidemiology studies [40-44] where the objective is to predict (interpolate) the value of a variable at unsampled locations in space and/or time, based on a sparse set of values observed at other locations. In these settings, geostatistical approaches have been shown to provide optimum prediction of unknown values in the sense of minimising prediction error variance whilst minimising prediction bias [39,45]. In simple terms, space-time geostatistical approaches exploit spatial and temporal autocorrelation in the variable of interest to predict missing or unsampled values as weighted linear combinations of data proximate in time and space. Weights are assigned to individual data that reflect the expected degree of similarity between points as a function of separation in space and time, with this relationship being formalised by a space-time covariance function estimated from observed data. Clustering between data points is taken into account and weights are calculated by solving a system of linear equations that minimises the error variance under the constraint of unbiasedness.

A protocol for using geostatistical techniques to compensate for incomplete HMIS data has been developed and tested recently [26,28] in which space-time kriging interpolation techniques [46-48] were used to 'fill the gaps' in the Kenyan HMIS database through space and time by predicting each missing monthly value where a facility had not reported. This approach exploited spatial and temporal autocorrelation present in the observed outpatient counts. Full details of this geostatistical analysis and variography have been presented previously [28]. Validation of this procedure found that estimates of national monthly and annual outpatient totals based on the geostatistically-completed database were accurate to within 4% and 2%, respectively, of the true values. Full details of the development, application, and validation of this geostatistical approach are presented elsewhere [26-28]. In the present study, we used this geostatistically-completed HMIS database to recalculate time series of mean all-cause outpatient cases per facility per month. These adjusted time series had two advantages over the plots based on the crudely-standardised HMIS data: (i) by incorporating predictions of missing data that took into account local patterns of outpatient attendance across space and through time, each estimated monthly or annual mean was more robust to the potential bias introduced by changing patterns of reporting between months; and (ii) a measure of the uncertainty introduced by the prediction of missing values (as derived from the validation procedure) was incorporated in the prediction of each monthly or annual mean value, and expressed as a 95% confidence interval. We calculated both crude and adjusted time series showing annual and monthly means for government-sector health facilities at the national and provincial level for each major facility type (dispensaries, health centres, and hospitals). These results were also disaggregated into malaria and non-malaria diagnoses. As a further comparison, we repeated the exercise for facilities from the faith-based sector that reported to the HMIS.

A source of potential inconsistency through time arises because the exact number of facilities in operation in any month is not known. Although a comprehensive list of government facilities was used in this study [29], information about when these facilities became operational was not available. The present study focuses on defining temporal trends in service use rather than absolute numbers of patients using services nationwide. We therefore included in the analysis only those facilities that reported (and were therefore operational) during the first year of the study period (1996).

## Results

### Data coverage and reporting rate

**Figure 1:**
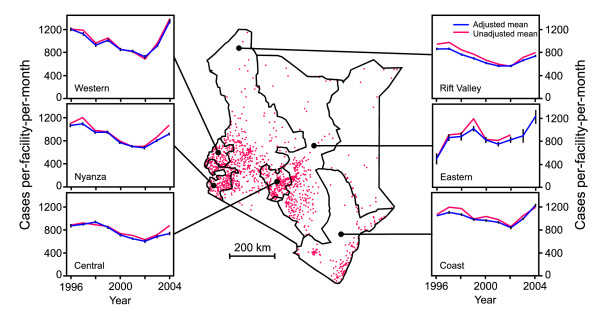
**Government sector outpatient cases (all diagnoses) by province**. Map of Kenya shows provincial boundaries and the distribution of 1 271 government health facilities included in this study (red dots). Plots are annual time series showing mean number of all-cause outpatient cases per facility per month at government health facilities in six provinces during 1996–2004. Unadjusted means were calculated directly from incomplete HMIS data. Adjusted means were based on a geostatistically-completed version of this dataset. Vertical bars on the adjusted mean plots show 95% confidence intervals.

Of the 2 421 government-run health facilities included in the national database, 1 271 reported data to the HMIS during 1996 (Figure 1) and were therefore selected for inclusion in this study (94 hospitals, 372 health centres, and 805 dispensaries). Within this set, the mean number of months reported by each facility was 54.2 (IQR 39–72) out of the full period of 108 months. The percentage of facilities reporting each month ranged from 9% in December 1997 to 75% in February 1996. A total of 62.4 million all-cause outpatient diagnoses were reported from the 1 271 facilities during the study period, with a mean of 906 cases per facility per month. The totals (monthly means) were 11.9 million (2 311) for hospitals, 19.9 million (947) for health centres, and 30.6 million (716) for dispensaries.

### Patterns of service use through time

**Figure 2:**
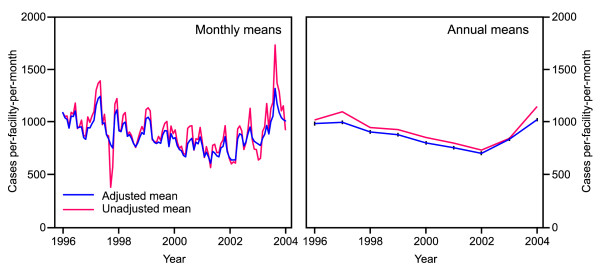
**Government sector outpatient cases (all diagnoses)**. Plots show adjusted and unadjusted annual and monthly time series of the mean number of all-cause outpatient cases per facility per month at government health facilities in Kenya during 1996–2004. Unadjusted means were calculated directly from incomplete HMIS data. Adjusted means were based on a geostatistically-completed version of this dataset. Vertical bars on the adjusted annual mean plot show 95% confidence intervals. The provenance and sensitivity of the HMIS data were affirmed by the observations of two marked aberrations in the monthly data: December 1997, a month of industrial action nationwide by nurses, and July 2004 when large publicity surrounded the reduction of user fees at government clinics.

Figure 2 shows crude and adjusted national time series of mean all-cause outpatient cases per facility per month for the government sector, presented by month and by year from 1996 to 2004. The annual time series shows a steady decline in outpatient use from an adjusted national mean (95% confidence interval) of 981 (974 to 987) cases per facility per month in 1996 to 699 (693 to 705) in 2002. This decline is then reversed in 2003 with a pronounced increase to 1 018 (1 013 to 1 024) cases per facility per month by 2004. The adjusted time series values are slightly lower than the crude values in each year. This effect is due to a tendency for larger, busier facilities to report more regularly meaning that the crude mean of reported records overestimates the true mean across all facilities, a bias that is addressed in the adjusted time series. The monthly time series reveals substantial within-year variation. Extreme monthly values are moderated in the adjusted series due to the smoothing effect of the space-time interpolation process. Seasonal variation occurs in each year, but the overall pattern of decline between 1996 and 2002 followed by an upturn during 2003 and 2004 remains pronounced. Furthermore, the monthly counts reveal two important temporal events: December 1997, a month of industrial action nationwide by nurses, and July 2004 when large publicity surrounded the reduction of user fees at government clinics.

**Figure 3:**
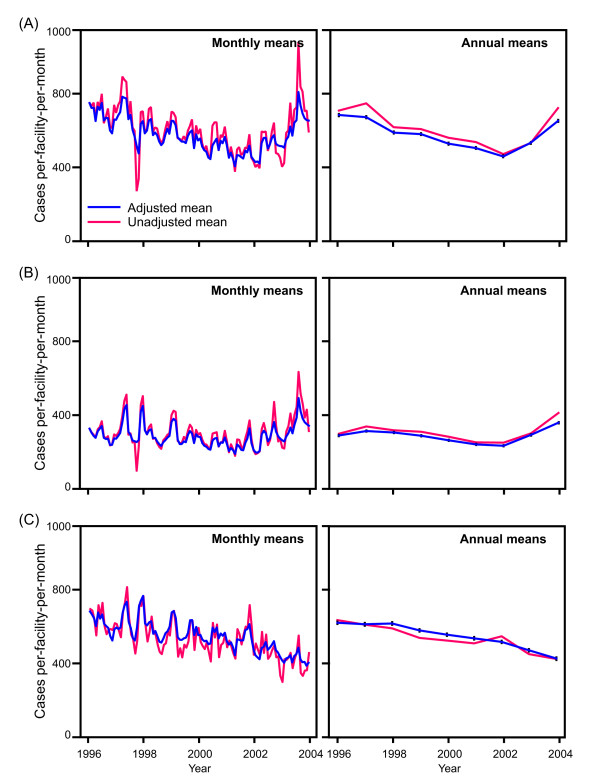
**Government sector non-malaria (A) and malaria outpatient diagnoses (B), and faith-based sector all-cause outpatient cases (C)**. Annual and monthly time series show mean values per facility per month at health facilities in Kenya during 1996–2004. Unadjusted means were calculated directly from incomplete HMIS data. Adjusted means were based on a geostatistically-completed version of this dataset. Vertical bars on the adjusted annual mean plots show 95% confidence intervals.

The same crude and adjusted annual time series were assembled at the provincial level (shown in Figure 1). Although there are variations between the provinces, the pattern of an overall decline up to 2002 followed by a sharp upturn is consistent across Central, Coast, Nyanza, Rift Valley, and Western provinces. The pattern for Eastern province is less consistent, although the number of available data in this province was substantially less than the others, with no data available in some months. Due to exceptionally low reporting rates, Nairobi and North Eastern province had insufficient data to obtain meaningful time series at the provincial level, and these plots are omitted. Time series were also produced separately for hospitals, health centres, and dispensaries at both national and provincial levels. These facility-specific time series followed identical patterns to those shown in Figure 2, excluding the possibility that the changes occurred due to use of higher or lower order health service providers (data not shown). To examine the influence of the dominant cause of outpatient diagnosis, malaria, we re-analysed the data for malaria cases and non-malaria cases separately (Figure 3). The similarities in the service use patterns for patients diagnosed as malaria compared to those not diagnosed as malaria suggest that the temporal service use patterns were not a disease-specific phenomenon.

The same statistical procedures were repeated for 402 faith-based sector health facilities that reported to the HMIS in 1996, providing a parallel time series to those developed for the government-run sector. Time series for these faith-based sector facilities (Figure 3) showed a similar decline in service use between 1996 and 2002 to that observed in the government-run sector. However, the sharp upturn witnessed in the government-run facilities in 2003 and 2004 was not present for the faith-based facilities, with service use continuing to decline.

## Discussion

We have used a robust geostatistical method to monitor changes in clinic use in Kenya over a 9-year time series reported by an imperfect national health information system dataset. By applying space-time geostatistical methods to minimize any statistical bias introduced by missing monthly data we were able to present, for the first time, reliable monthly and annual time series of the mean level of service use at the national and provincial level. Such output has immediate potential to enhance the capacity of decision makers in monitoring nationwide patterns of service use and assessing the impact of changes in health policy and service delivery.

By developing our approach for the case of Kenya during the 1996–2004 period, we have been able to reconstruct national service use patterns during a time of major changes in health policy and the resulting time series are able to reveal some striking features that are likely to be of direct interest to decision makers. Interpretation of these features serves to illustrate the potential of incomplete HMIS data, when handled appropriately, to detect important and policy-relevant changes in health service use. Of particular interest is the pattern of nationwide decline in service use between 1996 and 2002, followed by a sharp rise in the government-run sector beginning sometime during 2002. By 2004, annual service use in this sector had increased by approximately 45% compared to the nadir of 2002. The patterns observed at the national level were replicated sub-nationally, between different levels of the health service provision and whether malaria or non-malaria attendances were considered. Furthermore, the absence of any similar patterns among the faith-based sector in outpatient numbers during the same period suggests that the factors that stimulated the changes in the government-run clinics were specific to that sector.

The observed gradual decline in utilisation between 1996 and 2002 could be attributed to a general deterioration in the quality of government-run health services in terms of personnel, drugs and infrastructure, increases in user charges at government facilities, and a parallel growth in private commercial providers [49]. There could be several possible explanations for the sharp reversal of government clinic use in 2003 and 2004. It seems extremely unlikely that within such a short time period overall disease incidence would have increased by over 45% among the general population. Furthermore, the similarities between patterns for diagnoses of malaria, a vector-borne disease susceptible to short-term inter-annual variations, and the remaining non-malaria diagnoses suggest that the rise in service use in 2003–2004 was an indication of general health service use rather than a disease-specific change.

There have been several important changes in national health policy and services since 2002 that may have resulted in nationwide changes to service use behaviour. In mid-2004 there was a major change in user fee policy in Kenya with the introduction of the '10/20' initiative [50] that replaced an inconsistent system of widely varying fees with a standard fee of 10 Kenyan Shillings (KShs) at dispensaries and 20 KShs at health centres (equivalent to approximately 0.14 and 0.28 USD, respectively). This policy was widely adhered to in the early stages of its implementation and resulted in a significant net reduction in fees charged [50]. Increases in utilisation have been associated with the reduction or abolition of user fees in Uganda [51], South Africa [11], and Madagascar [52], and the abnormally high utilisation seen in our time series in July 2004, and higher average monthly utilisation in the last 6 months of 2004 compared with the first half of the year (Figure 2) seem consistent with this explanation. The inflexion point in utilisation occurs during 2002 – some time before the formal introduction of the 10/20 policy in mid 2004. The observed increases during this period correspond temporally to a series of political and health system changes: the arrival of a new government in December 2002, a substantial increase in Ministry of Health funding for essential drugs during 2003 [53], and the widespread media coverage in early 2003 of the Minister for Health's announcements that the government was committed to the provision of free malaria care treatment and a general abolition of user fees for vulnerable groups.

It is not the intention of this study to test formally different explanations for the various features revealed in our reconstructed time series. The results we present demonstrate that, contrary to the widely held perception, imperfect HMIS data can be used to monitor reliably a fundamental health-system metric: the extent to which a population is using health facilities. This monitoring can be implemented effectively at both national and provincial levels, and has sufficient sensitivity to detect both month-to-month variation and longer term trends. The use of a previously-developed geostatistical procedure that accounts for missing data allows the minimisation of bias in adjusted time series and the representation of uncertainty without the requirement of constructing detailed covariate datasets that are currently unavailable at the facility level.

There are at least three important caveats associated with the approach we present in this paper. Firstly, we address the critical problem of missing data and the confidence intervals we present account for the uncertainty introduced by the need to predict these missing data. We do not, however, address the inherent uncertainty of the data itself. We have assumed that, where a monthly record is present, the tally of outpatient visits is correct. The quality of HMIS data is known to vary widely, and the reliability of individual records cannot be quantified without substantial further studies or programmes to audit HMIS data quality. A second caveat arises from the need to limit our analysis to the cohort of facilities that were known to be operational at the beginning of the study (1996). Inevitably, the opening of new facilities may affect patient numbers at existing facilities and information to quantify the magnitude of this effect was not available. The most plausible influence of new facilities, if any exists, is the reduction of patient loads at existing ones. Such an effect would have exaggerated the observed decline in mean attendance levels between 1996 and 2002, but then mitigated the observed post-2002 increase. A third caveat is that the approach we present relies upon the availability of georeferencing information (latitude and longitude coordinates) for each facility [29], and such spatially referenced databases remain the exception in Africa. The need for such databases is becoming more widely recognised, however, and it is hoped that initiatives such as the World Health Organisation's Service Availability Mapping project [54] will increase their availability in the future.

## Conclusion

Many resource-constrained countries lack the evidence base for timely and effective health system decision-making, and this is exemplified by the scarcity of reliable data on health service use. Despite massive investments in HMIS across Africa, the poor data coverage of these systems has led to their gross under-use as an evidence base for decision-making at the national level. In this paper, we demonstrated an approach that enables incomplete HMIS data to be used to generate reliable information on changes in health systems, using the example of service use in Kenya. Specifically, the approach provided robust time series of mean levels of outpatient utilisation, with associated measures of uncertainty, that reveal for the first time substantial changes in service use over the last decade. Such information is of obvious utility to decision makers in monitoring nationwide patterns of service use and assessing the impact of changes in health policy and service delivery. The approaches presented in this paper will continue to provide a robust monitoring mechanism in Kenya, and serve as a template for other countries in the region with imperfect national data.

## Competing interests

The author(s) declare that they have no competing interests.

## Authors' contributions

PWG coordinated the study design, was responsible for the analysis and drafted/completed the manuscript. AMN, PMA, SIH and PG participated in the data assembly, analysis and drafting of the manuscript. SSK, CAG, SIH, PMA and RWS participated in the study design, analysis, interpretation of the findings and finalisation of the manuscript.

## Pre-publication history

The pre-publication history for this paper can be accessed here:


